# Soil organic carbon stocks in estuarine and marine mangrove ecosystems are driven by nutrient colimitation of P and N

**DOI:** 10.1002/ece3.2258

**Published:** 2016-06-26

**Authors:** Christian Weiss, Joanna Weiss, Jens Boy, Issi Iskandar, Robert Mikutta, Georg Guggenberger

**Affiliations:** ^1^ Institute of Soil Science Leibniz Universität Hannover Herrenhäuser Str. 2 D‐30419 Hannover Germany; ^2^ Department of Soil Science and Land Resources Bogor Agricultural University Kampus IPB Dramaga Bogor 16680 Indonesia; ^3^ Soil Science and Soil Protection Martin Luther Universität Halle Wittenberg Von‐Seckendorff‐Platz 3 D‐06120 Halle (Saale) Germany

**Keywords:** Ecosystem functioning, global change, Indonesia, marine and estuarine mangroves, nitrogen, phosphorus, soil organic carbon, stable isotopes

## Abstract

Mangroves play an important role in carbon sequestration, but soil organic carbon (SOC) stocks differ between marine and estuarine mangroves, suggesting differing processes and drivers of SOC accumulation. Here, we compared undegraded and degraded marine and estuarine mangroves in a regional approach across the Indonesian archipelago for their SOC stocks and evaluated possible drivers imposed by nutrient limitations along the land‐to‐sea gradients. SOC stocks in natural marine mangroves (271–572 Mg ha^−1^ m^−1^) were much higher than under estuarine mangroves (100–315 Mg ha^−1^ m^−1^) with a further decrease caused by degradation to 80–132 Mg ha^−1^ m^−1^. Soils differed in C/N ratio (marine: 29–64; estuarine: 9–28), *δ* ^15^N (marine: −0.6 to 0.7‰; estuarine: 2.5 to 7.2‰), and plant‐available P (marine: 2.3–6.3 mg kg^−1^; estuarine: 0.16–1.8 mg kg^−1^). We found N and P supply of sea‐oriented mangroves primarily met by dominating symbiotic N_2_ fixation from air and P import from sea, while mangroves on the landward gradient increasingly covered their demand in N and P from allochthonous sources and SOM recycling. Pioneer plants favored by degradation further increased nutrient recycling from soil resulting in smaller SOC stocks in the topsoil. These processes explained the differences in SOC stocks along the land‐to‐sea gradient in each mangrove type as well as the SOC stock differences observed between estuarine and marine mangrove ecosystems. This first large‐scale evaluation of drivers of SOC stocks under mangroves thus suggests a continuum in mangrove functioning across scales and ecotypes and additionally provides viable proxies for carbon stock estimations in PES or REDD schemes.

## Introduction

Mangroves are the biogeochemical interface between land and sea and therefore provide a multitude of services for both environments. They play an important role in coastal and reef protection (Alongi 2008; Koshiba et al. 2013) and provide indispensable nursery grounds for a plethora of species (Alongi 2002, 2008). Additionally, recent studies identified mangroves to be among the most carbon‐rich ecosystems (Donato et al. 2011; Kauffman et al. 2011; Murdiyarso et al. 2015), acting as a powerful sink for atmospheric carbon due to their high primary production (Twilley et al. 1992). Thus, ongoing pressure onto mangrove ecosystems by deforestation and degradation due to their increasing use for timber, firewood, and aquaculture (FAO 2007) imposes a high risk for the global climate, as mangrove loss is assumed to be responsible for 10% of total deforestation‐derived emissions worldwide (Donato et al. 2011). Additionally, mangroves are the source of >10% of the globally dissolved organic carbon (DOC) exported to the oceans (Jennerjahn and Ittekkot 2002; Dittmar et al. 2006). Thereby, mangrove loss already reduced carbon burial in the ocean by about 30 Tg year^−1^ (Duarte et al. 2005). These close interactions between the mangroves and the contiguous marine carbon cycle led to the coining of the term “blue carbon” to mainstream these aspects in the international policy discussion (e.g., Mcleod et al. 2011). The annual mangrove loss of 1–2% of the already reduced total area (Alongi 2002) causes a multitude of negative effects on livelihoods by the loss of mangrove‐related ecosystem services (Walters et al. 2008; Alongi 2011). This makes monitoring and management of mangrove carbon pools a prerequisite to benefit from compensatory financial instruments like Payment for Environmental Services (PES) and Reducing Emissions from Deforestation and forest Degradation schemes (REDD), in order to lower local vulnerability and to provide incentives for mangrove protection carried by local ownership. Regardless of political perspectives, soils are most decisive for the fate of carbon in mangroves, as they account for up to 98% of the total carbon stored in these ecosystems (Donato et al. 2011). This is underlined by recent studies revealing generally high amounts of soil organic carbon (SOC) as compared to terrestrial soils (Chmura et al. 2003; Donato et al. 2011, 2012; Kauffman et al. 2011).

Pedogenesis of mangrove soils differs in its processes between estuarine and marine mangrove ecosystems due to the different hydrological connection to the hinterlands, that is, whether a fluvial system contributes to the carbon pools by carbon‐containing sediment deposition or not. Donato et al. (2011) reported SOC stocks of estuarine mangroves to be substantially larger than those of marine (fringe) mangroves, but this comparison has to be regarded cautiously as soil columns of varying depths (1–2 m) were taken into account and a standardization to a defined depth is needed if not the whole soil profile is available for comparison. In contrast to this, carbon concentrations in soil of marine mangroves (0.061 g C cm^−3^) were found considerably higher than those of estuarine mangroves (0.038 g C cm^−3^, Donato et al. 2011). These differences may be explained by riverine sedimentation diluting autochthonous carbon sources (e.g., litter) by allochthonous material. But Breithaupt et al. (2014) clearly showed that the burial of organic carbon and therefore the sedimentation does neither correlate with SOC concentration nor SOC stocks, as hypothesized before (Kristensen et al. 2008; Breithaupt et al. 2012). Hence, different SOC turnover between the two mangrove traits, as known from other wetland ecosystems (Lugo and Snedaker 1974; Brinson et al. 1981), must be taken into account. Important factors inhibiting the decomposition of organic matter is its quality and the availability of nutrients, such as nitrogen (N) and phosphorus (P), to the decomposing microbial community.

Indeed, mangrove litter is often of poor quality exhibiting very high C/N ratios >200 (Rao et al. 1994), which imposes severe decomposition obstacles to most microorganisms. Initial decomposition of litter in estuarine mangroves was found to happen within weeks, often mediated by ground‐dwelling crabs (Nordhaus et al. 2006), but this decomposition still ends up with high C/N ratios of around 40 (Bosire et al. 2005). Such high C/N ratios suggests N as the nutrient potentially limiting mangrove growth (Reef et al. 2010), which may be overcome by symbiotic N_2_ fixation, covering the larger part of N demand in many mangrove ecosystems (Sengupta and Chaudhuri 1991; Holguin et al. 2001; Bashan and Holguin 2002; Reef et al. 2010). Estimation of litter quality is offered by comparing the *δ* ^13^C values of leaves and soil (e.g., Xia et al. 2015), as is the contribution of nitrogen‐fixing symbionts to N‐nutrition by its *δ* ^15^N values (Inglett et al. 2011).

As another element, P has been identified as the limiting nutrient in many mangrove ecosystems (e.g. Lovelock et al. 2007; Reef et al. 2010). Fertilization experiments have shown that the main limiting nutrient (N or P) can vary on relatively small gradients. Feller et al. (2002) concluded that fringe mangroves directly prone to sea rather tend to be N limited, whereas hinterland‐oriented fringe mangroves tend to be P limited with possible colimitation of both nutrients in the transition zone. This concept is in match with the one of generally P‐limited terrestrial tropical ecosystems (Vitousek 1984) and rather N limited marine systems (Howarth and Marino 2006). Along a sea‐to‐land gradient, this different nutrient limitation might alter organic matter decomposition and with that SOC storage. According to Feller et al. (2002), P fertilization leads to increased soil organic matter decomposition in all examined positions of mangroves along this sea‐to‐land gradient and deduced that seaward‐oriented mangroves underlie higher decomposition rates than hinterland‐oriented mangroves. This contradicts the larger SOC concentrations in soil of marine mangroves (cf. seaward‐oriented mangroves on a small scale) as compared to estuarine mangroves (cf. hinterland‐oriented mangroves on a small scale) as was reported by Donato et al. (2011). A possible explanation for this contradiction might lie in the difference in scale and functional trait of mangroves in the way that, for example, a marine mangrove differs in its biogeochemical functioning from an estuarine mangrove. Thus, the question remains whether a general driving factor exists which modulates SOC stocks in mangrove soils irrespective of its marine or estuarine nature.

To clarify this, we conducted a biogeochemical survey along the sea‐to‐land gradient on regional scale, spanning three contrasting mangrove ecosystems in Indonesia comprising marine and estuarine mangroves in different states of degradation. We hypothesize that the amount of SOC stored in mangrove soil is a function of the interplay between the mangrove's position along the land–sea gradient and the thereby resulting nutrient gradient, which is affecting the quality and decomposability of organic matter produced and recycled by the species adapted to the respective situation.

## Materials and Methods

### Study sites and sampling scheme

**Figure 1:**
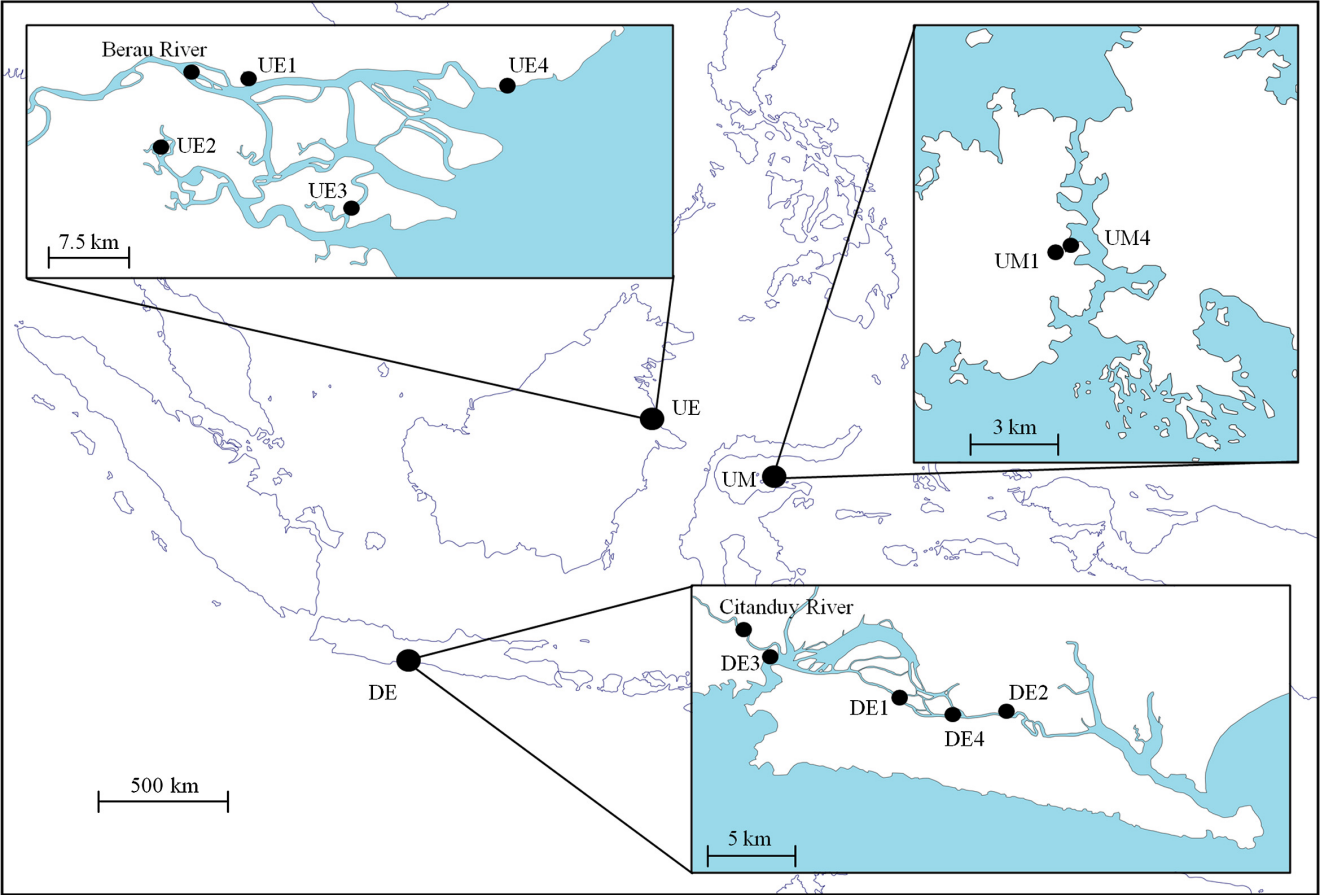
Sample sites of different mangrove settings in Indonesia. In southern Java, Segara Anakan Lagoon (DE), and in eastern Kalimantan, the Berau estuary (UE) was sampled. Mangrove sites under absence of estuarine influence were sampled at the Togian Islands, Sulawesi (UM) were sampled. UM2 and UM3 are in between UM1 and UM4 but not shown for scaling reasons. Abbreviations denote: DE, degraded estuarine mangroves; UE, undegraded estuarine mangroves; UM, undegraded marine mangroves.

**Figure 2:**
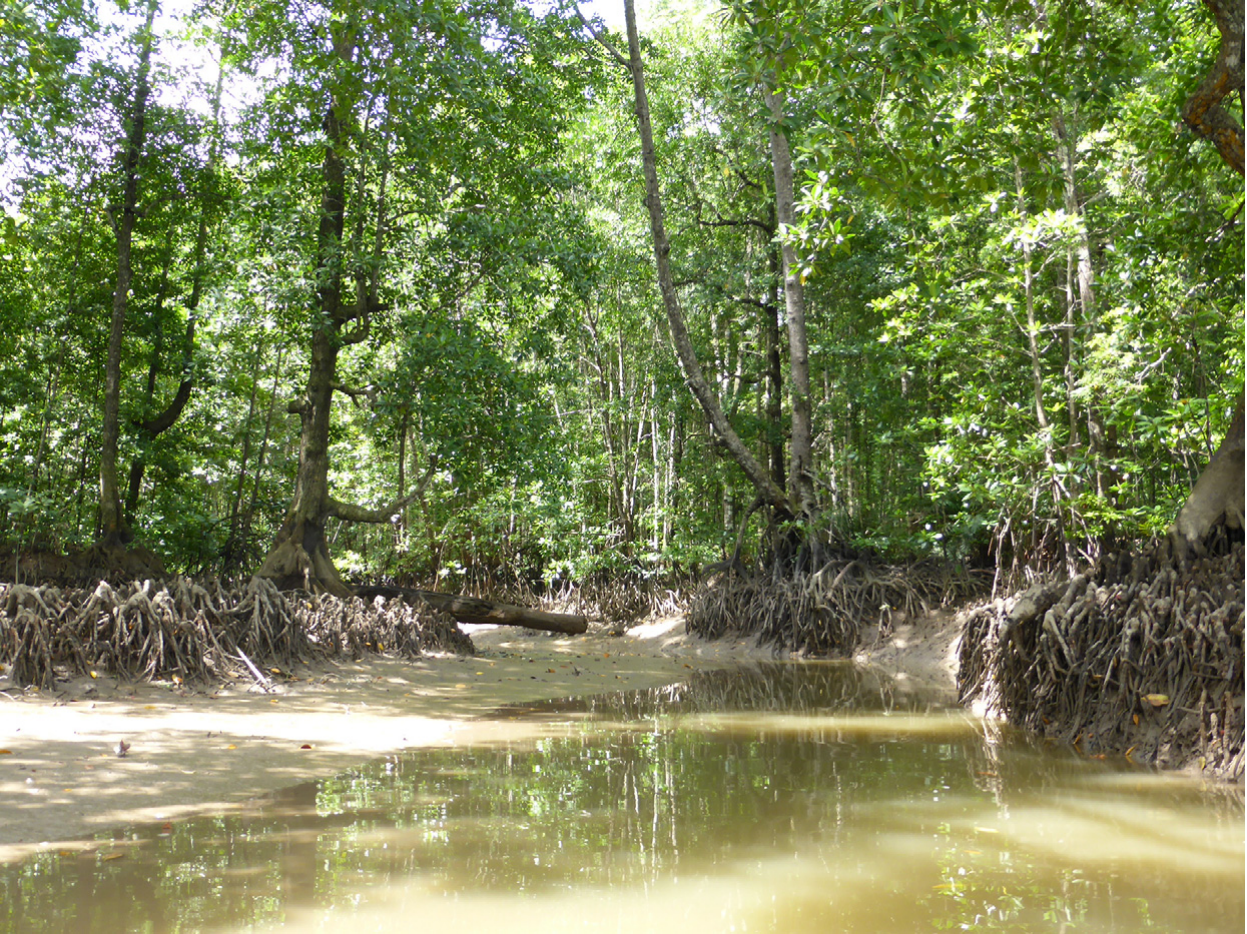
Typical tidal channel in an undegraded estuarine mangrove. Photograph was taken in the central part of the Berau estuary.

To ensure the requirements of a large‐scale study, the study sites are distributed over Indonesia with distances of several hundred kilometers in between (Fig. 1). Indonesia was chosen because it is the most mangrove‐rich country with high rates of mangrove loss, but nevertheless still providing a high variability of different mangrove ecosystems (Giri et al. 2011). The study sites comprise three major mangrove types as estuarine mangroves of degraded and undegraded state and undegraded marine mangroves were objects of this study. As an example, a typical tidal channel of an undegraded estuarine mangrove is shown in Figure 2. An overview of all sampled stations is given in Table 1. The Segara Anakan Lagoon in southern Central Java was chosen as a representative case for degraded estuarine mangroves (DE). The lagoon, into which the Citanduy River discharges, was once covered by a dense mangrove forest, but severe deforestation, hinterland erosion, intensive agricultural use of the hinterland, and industry in the eastern parts of the lagoon led to the prevailing degraded situation (Yuwono et al. 2007). Nowadays, the lagoon's vegetation is affected by shrubby halophytes (*Derris trifoliata* and *Acanthus ilicifolius*) and a mixture of small regrown mangrove trees of different species which are regularly cleared long before reaching tree size. Therefore, we regarded this mangrove ecosystem as heavily degraded. Due to the patchy cover of vegetation, a successional vegetation gradient from the seaward edge to the hinterland could not be observed. Hence, sampling was carried out at four different vegetation patterns representative for this lagoon: (1) areas only covered by *Derris trifoliata* and *Acanthus ilicifolius* (DE1); (2) areas covered by *Nypa fruticans* and *Derris trifoliata* (DE2); (3) younger mixture (average age 4 years) of different mangrove species (DE3); and (4) older mixture (average age 6 years) of different mangrove species (DE4).

**Table 1:** General overview of all sampled locations and their corresponding geographical position

Station/replicate	Description	Latitude	Longitude
*Degraded estuary*			
Citanduy River	Nonmangrove riverine sediment sampling location		
1, 2, 3		S 07° 39.58′	E 108° 47.11′
DE1	Degraded estuarine mangroves dominated by *Derris trifoliata* and *Acanthus ilicifolius*		
1		S 07° 41.79′	E 108° 51.60′
2		S 07° 41.80′	E 108° 51.81′
3		S 07° 42.11′	E 108° 52.04′
DE2	Degraded estuarine mangroves dominated by *Nypa fruticans* and *Derris trifoliata*		
1		S 07° 42.51′	E 108° 55.22′
2		S 07° 42.50′	E 108° 54.80′
3		S 07° 42.42′	E 108° 54.50′
DE3	Degraded estuarine mangroves, vegetated by a mixture of 4‐year‐old regrown mangroves		
1		S 07° 40.67′	E 108° 47.60′
2		S 07° 40.68′	E 108° 47.59′
3		S 07° 40.66′	E 108° 47.61′
DE4	Degraded estuarine mangroves, vegetated by a mixture of 6‐year‐old regrown mangroves		
1		S 07° 42.51′	E 108° 53.18′
2		S 07° 42.54′	E 108° 53.37′
3		S 07° 42.56′	E 108° 53.58′
*Undegraded Estuary*			
Berau River	Nonmangrove riverine sediment sampling location		
1, 2, 3		N 02° 11.46′	E 117° 39.63′
UE1	Transition from mangroves to nonmangroves		
1		N 02° 11.08′	E 117° 42.52′
2		N 02° 11.31′	E 117° 42.70′
3		N 02° 11.23′	E 117° 42.61′
UE2	Landward undegraded estuarine mangroves		
1		N 02° 08.71′	E 117° 37.22′
2		N 02° 07.14′	E 117° 37.72′
3		N 02° 07.06′	E 117° 36.62′
UE3	Central undegraded estuarine mangroves		
1		N 02° 03.47′	E 117° 48.58′
2		N 02° 02.80′	E 117° 48.40′
3		N 02° 01.92′	E 117° 48.15′
UE4	Seaward undegraded estuarine mangroves		
1		N 02° 11.46′	E 117° 58.76′
2		N 02° 10.35′	E 117° 55.67′
3		N 02° 08.21′	E 117° 56.66′
*Undegraded Marine Mangroves*			
UM1	Landward undegraded marine mangroves		
1		S 00° 23.30′	E 122° 03.16′
2		S 00° 23.31′	E 122° 03.17′
3		S 00° 23.30′	E 122° 03.17′
UM2	Central undegraded marine mangroves		
1, 2, 3		S 00° 23.20′	E 122° 03.38′
UM3	Seaward undegraded marine mangroves		
1, 2, 3		S 00° 23.15′	E 122° 03.53′
UM4	Transition from mangroves to seaweed		
1, 2, 3		S 00° 23.14′	E 122° 03.55′

For undegraded natural estuarine mangroves (UE), the in vast parts only little anthropogenically degraded Berau estuary in eastern Kalimantan was chosen. The Berau estuary ranges roughly 40 km upstream and has a maximum width of approximately 25 km. Aquacultures for shrimp farming occur, but this kind of land‐use is in early stage. The catchment area of the Berau River roughly corresponds to the Berau Regency, except its south‐eastern part. The hinterland is dominated by tropical rainforest which was partially cleared for palm oil plantation, open pit coal mining, or settlement area. Within the estuary, a gradient from the hinterland to the shoreline was sampled. (1) As a transitional stage between mangroves and hinterland, soil samples from a small freshwater tributary were taken. This plot was dominated by nonmangrove vegetation, although isolated plants of *Sonneratia sp*., *Nypa fruticans*, and *Acanthus ilicifolius* were found (UE1). (2) The second hinterland‐orientated site underlies far less fresh water influence because it is located in a tidal channel being connected to the sea but receiving no direct input from the river. Vegetation was entirely constituted by mangrove species, with *Bruguiera sexangula*,* Rhizophora stylosa*, and *Xylocarpus granatum* dominating (UE2). (3) Closer to the sea and at the central part of the estuary, *Bruguiera sexangula* and *Rhizophora apiculata* were dominating (UE3). (4) The most seaward station was sampled in the direct vicinity to the principal branch of the estuary. Only mangrove species were observed with dominance of *Sonneratia alba* (UE4).

Natural undegraded marine island mangroves (UM) were sampled on the Togian Islands. These Islands are located in the Gulf of Tomini off the coast of Central Sulawesi. Due to the lack of rivers, these mangroves underlie marine conditions without being influenced by the hinterland except occasionally occurring surface run‐offs after storm events. The mangroves on the Togian Islands were found under pristine conditions. Logging for aqua cultural use or timber could not be observed. Sampling was carried out on a four station gradient from the hinterland to the seaward edge with UM1 being the most landward and UM4 the most seaward site. At the most landward station (UM1), *Bruguiera* sp. was dominating with only a few trees of *Rhizophora* sp., and at UM2, a mixture of *Bruguiera* sp. and *Rhizohora* sp. were found, whereas at UM3 (seaward station), only *Rhizophora* sp. could be observed. At UM4, the outer rim of the mangrove belt with beginning colonization of *Rhizophora* sp. by stolons, seagrass is in dominance.

Tidal data were acquired based on the program “WXtide32” for tide prediction. Mean tidal ranges were calculated as 1.75 m for the undegraded estuary (0.5 m during neap tide to 3 m during spring tide), 1 m for the degraded estuary (0.5 m during neap tide to 1.5 m during spring tide), and 0.75 m for the marine mangroves (0.5 m during neap tide to 1 m during spring tide). Results modeled by this program can be regarded as validated because another study measured comparable values for the Segara Anakan lagoon (Holtermann et al. 2009).

### Sampling

Soil samples were taken at all plots in the intertidal zone during low tide using a custom made soil corer of 3.7 cm diameter. Potential compression of the soil cores was taken into account by scaling the cores to the drill depth and the inner diameter of the corer. The maximum sampling depth was 3 m which was reached in case of all sites in Segara Anakan (DE) and the most landward station at the Togian Islands (UM1). The Berau estuary was sampled with a maximum depth of 2 m in case of UE2 and UE3 and 1 m in case of UE1 and UE4. Depth increments for sampling were 0.5 m in case of DE and UE and 0.2 m in case of UM. Each location was sampled with three replicates. In case of both gradients (UE and UM), replicates were chosen randomly in a few tens of meters next to each other. In case of vegetation pattern‐based sampling at DE, replicates were scattered randomly within each uniform vegetation pattern with distances of up to several hundred meters between the replicates (Table 1). All samples were air‐dried to avoid any alteration during transport and storage. Plant samples were taken randomly from the most abundant species with distinction of root and leaf samples. Like soil samples, plant samples were air‐dried already in the field.

### Processing of samples

Air‐dried soil samples were gently crushed to destroy drying‐induced aggregates and to enable subsequent sieving to remove coarse organic material like larger parts of fresh roots. Sieving was conducted with 8‐mm mesh size and had no influence on the texture of the soil because the coarsest fraction found in all samples was sand with neither gravel nor stones present.

Organic carbon (OC), total nitrogen (TN), *δ* ^13^C, and *δ* ^15^N were measured with an elemental analyzer combined with an isotope ratio mass spectrometer (EA‐IRMS; Isotope Cube^®^, Elementar Analysensysteme GmbH, Hanau, Germany, linked to Isoprime Mass Spectrometer^®^, Isoprime Ltd., Cheadle Hulme, U.K.). If necessary (i.e., at UE4), carbonates were removed by fumigation with HCl after Harris et al. (2001). Bulk density was calculated by means of soil corer volume and the dry weight of the sample. Carbon stocks were calculated based on SOC concentration and bulk density, for the different depth increments.

Water‐extractable nutrients were determined by water extraction: 10 g of dry soil sample was extracted with 50 mL of deionized water (18 MΩ cm^−1^). Subsequently, water extracts were filtered <0.45 *μ*m using polyethersulfone membrane filters (Supor^®^‐450, Pall Life Sciences, Port Washington, NY) and measured with inductively coupled plasma in combination with optical emission spectroscopy (ICP‐OES; Varian 725‐ES, Varian Inc. Palo Alto, CA) for water‐extractable P. NO_3_‐N and NH_4_‐N were measured in the same extracts with a continuous flow analyzer (CFA; San^++^, Skalar Analytical B.V., Tinstraat 12, 4823 AA, Breda, the Netherlands). Total P was determined by muffling 1 g of soil sample (2 h ramp with 250°C h^−1^ followed by 4 h at 500°C), and subsequent extraction with 10 ml: 1.0 mol L^−1^ HCl and a dilution of 1:5 with deionized water. Corresponding to the water extracts, acid extracts were filtered at <0.45 *μ*m and measured for P by ICP‐OES.

The conventional radiocarbon age was estimated based on ^14^C measurements by accelerator mass spectroscopy (AMS; 3MV Tandetron Accelerator, HVEE, Amersfoort, the Netherlands) of a small amount of seven samples from the undegraded estuary and the undegraded marine mangroves (UE and UM). Due to regularly occurring disturbances in the degraded estuary (DE), these samples were not taken into account. Samples from the undegraded estuary (UE2, UE3, UE4; each bulked from three replicates) originate from the depth increment of 50 to 100 cm, and samples from the undegraded marine mangroves (UM1, UM2, UM3, UM4; each bulked from three replicates) originate from the depth increment of 60 to 100 cm.

## Results

**Figure 3:**
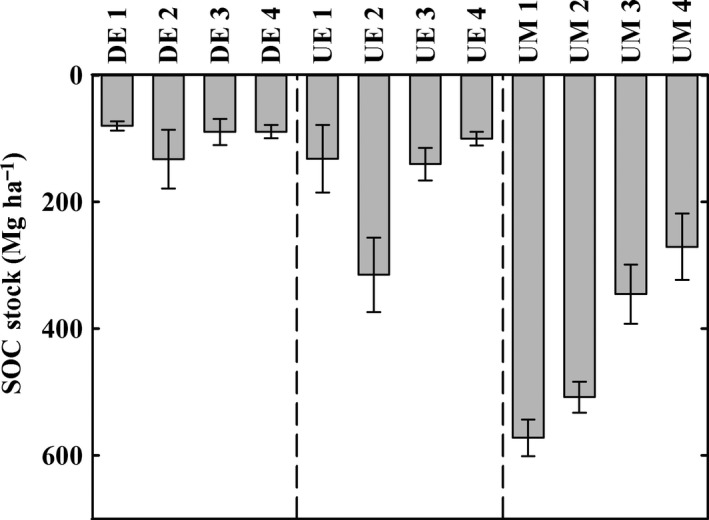
Soil organic carbon stocks of all stations of the topmost meter of mangrove soil. Pictured are the three different mangrove settings with DE = degraded estuary (Segara Anakan), UE = undegraded estuary (Berau estuary), and UM = undegraded marine mangroves (Togian Islands). All stations are denoted as described in Table 1. Mean values of *n* = 3 replicates are plotted. Error bars indicate standard deviation.

**Figure 4:**
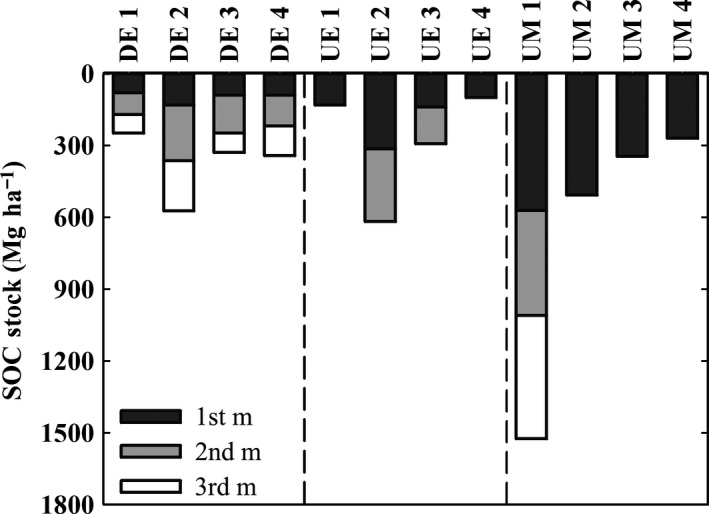
Soil organic carbon (SOC) stocks of all stations of all sampled depths. Sampling was limited to a maximum depth of 3 m due to sampling technique or minor sediment thickness. Available data indicate possible sampling depth. Homogenous distributed SOC stocks within each core are evident. All stations are denoted as described in Table 1.

The textures of the mangrove soils from the estuaries (DE, UE) vary over wide ranges with compositions between silt loam, sandy loam, and clay in case of DE and compositions between silt loam, loamy sand, and silty clay in case of UE. Due to their high amount of organic matter, the soils from the undegraded marine mangroves (UM) cannot be described like the mineral soils (DE, UE). Therefore, their texture is simply characterized as “organic” (Table 2). Bulk densities of the estuarine mangrove soils, which are mineral soils of different textures, were higher (DE: 0.33–0.80 g cm^−3^; UE: 0.42–0.67 g cm^−3^) than bulk density of the high‐organic soils from marine mangroves (UM: 0.18–0.27 g cm^−3^; Table 2). In contrast to this, OC concentrations were the largest in marine mangrove soils (UM: 172.6–262.4 mg g^−1^), intermediate in undegraded estuarine mangrove soils (UE: 15.3–85.1 mg g^−1^), and smallest in degraded estuarine mangrove soils (10.7–46.0 mg g^−1^, Table 2). As a result of this, SOC stocks show considerable differences between the three main mangrove types DE, UE and UM (Fig. 3). Maximum OC stocks within the topmost meter of mangrove soils were found in the marine island mangroves (UM) with approximately 570 Mg ha^−1^. This is almost twice the amount of the maximum SOC stock of the undegraded estuarine mangrove sites (UE, approx. 310 Mg ha^−1^) and roughly threefold the amount of the maximum SOC stocks of the degraded estuarine sites (DE). Organic C stocks of soil samples taken deeper than 1 m exhibited the same relative differences (Fig. 4). In total, soils of marine mangroves stored considerably more OC per soil volume than estuarine mangroves. Additionally, we observed a land‐to‐sea gradient for undegraded mangrove ecosystems, no matter if marine or estuarine, with higher SOC stocks toward the inland in both cases (Fig. 4).

**Table 2:** Overview of measured soil parameters. Denoted values are mean values of *n* ≥ 3; standard deviation is stated in parentheses. OC, C/N, *δ* ^13^C, and *δ* ^15^N were measured by EA‐IRMS. P_total_ was measured on HCl extracts after dry ashing by ICP‐OES. Water extracts (10 g soil: 50 mL H_2_O) were used to measure P_water‐extractable_, and NH_4_‐N. P_water‐extractable_ was measured by ICP‐OES, NH_4_‐N by CFA. NO_3_‐N was measured as well but values were below detection limit and therefore not shown

Station/Depth [cm]	Texture [US‐Soil‐Taxonomy]	Bulk Density [g cm^−3^]	OC [%]	C/N [‐]	*δ* ^13^C [‰ PDB]	*δ* ^15^N [‰ air]	P_total_ [mg kg^−1^]	P_water‐extractable_ [mg kg^−1^]	NH_4_‐N [mg kg^−1^]
*Degraded estuary*									
Citanduy River									
0–100	Silt loam/loam	0.69 (0.11)	1.08 (0.27)	8.84 (0.34)	−25.61 (0.56)	4.17 (0.26)	196.91 (2.32)	0.49 (0.42)	22.22 (8.34)
DE1									
0–100	Clay	0.41 (0.03)	2.18 (0.30)	9.89 (0.95)	−26.96 (0.51)	4.02 (0.47)	177.52 (32.51)	0.23 (0.18)	14.72 (1.81)
100–200	Clay	0.61 (0.06)	1.69 (0.24)	10.90 (1.43)	−26.57 (0.37)	4.36 (0.52)	n.a.	0.28 (0.24)	6.37 (1.59)
200–300	Clay	0.59 (0.07)	1.26 (0.06)	10.20 (0.31)	−25.68 (0.19)	4.18 (0.21)	n.a.	0.19 (0.13)	6.38 (1.29)
DE2									
0–100	Clay	0.33 (0.06)	3.84 (0.99)	16.26 (6.23)	−27.65 (0.26)	3.46 (0.66)	220.63 (12.84)	0.86 (0.60)	11.19 (5.11)
100–200	Clay	0.57 (0.06)	4.60 (0.77)	22.97 (2.08)	−27.96 (0.37)	3.02 (0.37)	n.a.	1.38 (0.20)	11.42 (5.21)
200–300	Sandy loam/sandy clay loam						n.a.		
DE3									
0–100	Silty clay	0.47 (0.09)	1.57 (0.32)	13.83 (3.25)	−27.82 (0.63)	2.91 (0.16)	181.80 (32.37)	0.12 (0.11)	5.44 (1.66)
100–200	Silty clay	0.72 (0.12)	2.38 (1.44)	16.80 (2.44)	−27.47 (0.73)	3.02 (0.35)	n.a.	0.56 (0.39)	7.09 (0.97)
200–300	Silty clay	0.80 (0.02)	1.07 (0.13)	10.94 (1.03)	−25.94 (0.43)	3.17 (0.29)	n.a.	0.07 (0.04)	4.24 (0.58)
DE4									
0–100	Clay	0.39 (0.05)	2.37 (0.10)	13.01 (0.64)	−27.84 (0.16)	3.59 (0.33)	149.24 (10.89)	0.53 (0.51)	8.15 (1.80)
100–200	Clay	0.56 (0.06)	2.34 (0.72)	14.48 (2.50)	−27.64 (0.18)	3.76 (0.25)	n.a.	0.37 (0.25)	6.36 (0.59)
200–300	Clay	0.53 (0.05)	2.28 (0.55)	14.43 (2.25)	−27.42 (0.32)	4.14 (0.47)	n.a.	0.39 (0.22)	6.29 (1.37)
*Undegraded Estuary*									
Berau River									
0–100	Silt loam	0.60 (0.04)	3.24 (0.08)	13.33 (0.29)	−29.50 (0.07)	2.53 (0.05)	161.21 (0.13)	0.15 (0.01)	34.66 (1.46)
UE1									
0–100	Silty clay/silty clay loam	0.44 (0.07)	3.60 (0.20)	13.08 (0.15)	−29.59 (0.32)	3.04 (0.59)	218.70 (16.95)	0.30 (0.18)	24.76 (12.23)
UE2									
0–100	Silty clay	0.45 (0.17)	8.51 (3.46)	28.15 (5.50)	−28.14 (0.40)	3.72 (1.31)	162.13 (20.03)	1.16 (0.68)	9.19 (3.28)
100–200	Silty clay	0.59 (0.17)	5.30 (2.98)	24.38 (5.88)	−28.11 (0.44)	4.01 (1.64)	n.a.	1.00 (0.70)	9.24 (3.16)
UE3									
0–100	Silty clay	0.42 (0.06)	3.34 (0.78)	14.37 (2.93)	−28.99 (0.10)	6.31 (2.42)	210.27 (42.97)	1.18 (0.43)	10.71 (3.15)
100–200	Silty clay	0.61 (0.05)	2.79 (0.40)	13.11 (2.46)	−28.67 (0.24)	7.24 (2.02)	n.a.	1.09 (0.65)	10.93 (2.86)
UE4									
0–100	Loamy sand/sandy clay loam/silty clay	0.67 (0.26)	1.53 (0.61)	13.74 (2.15)	−28.50 (0.35)	3.20 (0.97)	246.76 (57.52)	0.63 (0.21)	7.89 (6.23)
*Undegraded Marine Mangroves*									
UM1									
0–100	Organic texture	0.27 (0.11)	22.89 (0.34)	42.10 (6.23)	−27.96 (0.58)	−0.16 (0.23)	237.66 (60.85)	6.30 (1.08)	43.75 (2.40)
100–200	Organic texture	0.18 (0.02)	26.24 (2.47)	51.27 (6.04)	−27.85 (0.27)	−0.40 (0.41)	n.a.	4.07 (0.99)	43.62 (9.92)
200–300	Organic texture	0.23 (0.02)	25.06 (0.77)	64.36 (7.21)	−27.62 (0.50)	−0.25 (0.31)	n.a.	2.53 (0.83)	38.67 (2.86)
UM2									
0–100	Organic texture	0.23 (0.05)	22.43 (2.45)	35.53 (5.34)	−27.92 (0.38)	−0.06 (0.24)	261.61 (70.81)	5.74 (1.47)	41.73 (10.53)
UM3									
0–100	Organic texture	0.19 (0.01)	17.84 (0.44)	28.55 (4.09)	−27.60 (0.18)	0.25 (0.20)	344.99 (21.59)	2.50 (1.07)	33.96 (1.88)
UM4									
0–100	Organic texture	0.19 (0.03)	17.26 (0.28)	29.01 (1.36)	−27.62 (0.12)	0.72 (0.57)	328.00 (n.a.)	2.32 (1.03)	31.79 (2.22)

**Figure 5:**
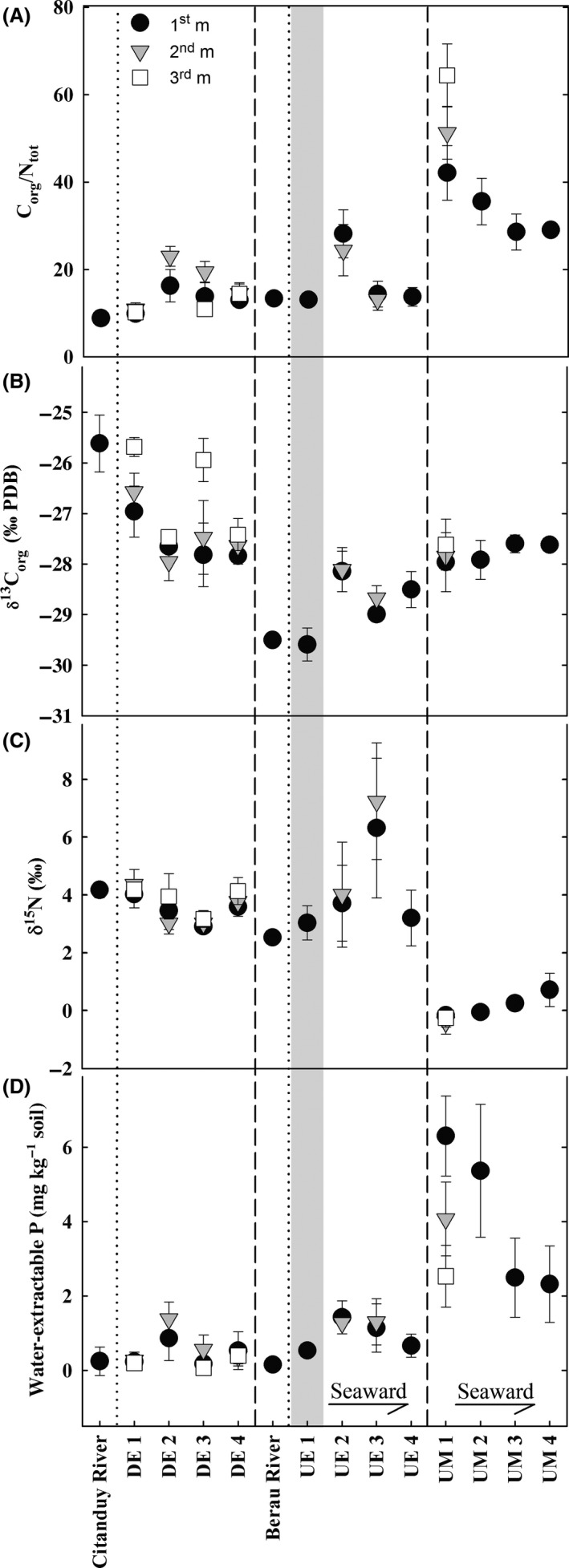
C/N ratios (a), *δ* ^13^C (b), *δ* ^15^N (c), and water‐extractable P concentrations (d) of soils of all stations and river sediments of discharging rivers in case of the estuaries. All stations are denoted as described in Table 1. Please note that station UE1 is within the transition zone to nonmangrove hinterland vegetation and therefore displayed on gray background. Mean values of *n* = 3 replicates are plotted. Error bars indicate standard deviation.

Leaves and roots of mangrove species showed contrasting C/N ratios for arboreal mangroves (leaves 33–81, roots 36–143), and the shrubby halophytes invading degraded mangroves (e.g., leaves of *Acanthus ilicifolius* and *Derris trifoliata*, both around 19; Table 3). The major differences in the C/N ratios of the soils appeared between marine mangroves (UM) and estuarine mangroves (UE, DE; Fig. 5A). Soils of marine mangroves had far higher C/N ratios than those of estuarine mangroves. Differences between the degraded and the undegraded estuaries were negligible and showed no clear trend. Nevertheless, the highest C/N ratios in estuarine mangrove soils were found under undegraded mangroves (UE2). In estuarine mangroves soils, the C/N ratio did not vary with soil depths, whereas in marine mangrove soils, C/N ratio increased with depth (Fig. 5A).

**Table 3:** C/N ratios and *δ* ^13^C values of different mangrove plant species

Species	C/N		*δ* ^13^C (‰ PDB)	
	Fresh leaves	Fresh roots	Fresh leaves	Fresh roots
*Rhizophora stylosa*	54.1	108.9	−29.74	−29.10
*Rhizophora apiculata*	36.9	143.2	−30.35	−27.96
*Bruguiera parviflora*	81.2	71.1	−28.83	−28.58
*Bruguiera sexangula*	51.9	92.8	−32.70	−28.45
*Xylocarpus granatum*	41.3	96.2	−31.15	−28.29
*Sonneratia alba*	32.6	62.7	−30.65	−28.09
*Aegiceras corniculatum*	70.0	92.8	−27.59	−28.04
*Nypa fruticans*	52.9	35.8	−25.41	−25.86
*Derris trifoliata*	19.3	n.a.	−28.33	n.a.
*Acanthus ilicifolius*	18.8	n.a	−26.32	n.a.

The *δ* ^13^C values of the examined mangrove plants as litter source ranged from −33 to −25‰ for leaves and from −29 to −26‰ for roots with no differences between arboreal mangroves and shrubby halophytes observable (Table 3). Marine mangrove soils showed *δ* ^13^C values close to −28‰ with little variation. Unlike this, soil *δ* ^13^C values of estuarine mangrove soils varied more, ranging from −28‰ to −25‰ in the degraded estuary mangrove and from −30‰ to −28‰ in the undegraded one. In case of the degraded estuary, *δ* ^13^C increased with depth at DE1 and DE3. All other stations did not show any depth dependences (Fig. 5B). More pronounced differences were found in terms of *δ* ^15^N between marine and estuarine mangrove soils. While marine mangrove soils had *δ* ^15^N of −0.6 to 0.7‰, estuarine mangrove soils exhibited clearly positive values of 2.5 to 7.2‰ (Fig. 5C). No significant differences could be observed between undegraded and degraded estuarine mangrove soils, although *δ* ^15^N values spanned a wider range in the former.

Water‐extractable P of the mangrove soils differed significantly between marine and estuarine mangroves with; again, no differences between the both estuarine types (Fig. 5D). Marine mangrove soils exhibited the largest amounts of water‐extractable P, ranging from 2.3 to 6.3 mg kg^−1^ soil, whereas estuarine mangrove soils showed comparably small concentrations <2 mg kg^−1^ soil (Fig. 5D). Total P content of soils differed between marine and estuarine mangroves, ranging from 238 to 345 mg kg^−1^ in marine‐ and 162 to 247 mg kg^−1^ in estuarine mangrove soils. No differences were observed between degraded and undegraded estuarine soils (Table 2).

The NO_3_‐N concentration of the soils was below detection limit in all soils, whereas the NH_4_‐N concentration revealed differences between the different mangrove ecosystems. NH_4_‐N concentrations were lowest in soils of the degraded estuary (5.4–14.9 mg kg^−1^), intermediate in soils of the undegraded estuary (8.0–34.8 mg kg^−1^), and highest in the marine mangrove soils (31.9–43.9 mg kg^−1^, Table 2).

The mean conventional radiocarbon age of the samples from the undegraded estuary was 350 ± 315 years B.P. (UE2: 633 ± 30 years B.P.; UE3: 406 ± 23 years B.P.; UE4: 10 ± 25 years B.P.), whereas the mean age of the undegraded marine counterparts was younger averaging 69 ± 110 years B.P. (UM1: 231 ± 25 years B.P.; UM2: recent; UM3: 46 ± 2 years B.P.; UM4: recent).

## Discussion

The pronounced differences between marine and estuarine SOC stocks are well in accordance to those of other studies at smaller scale. Kauffman et al. (2011) found seaward‐oriented marine mangrove soils (cf. UM3, UM4) to store 354–377 Mg ha^−1^ m^−1^, interior marine mangrove soils (cf. UM2) 380–424 Mg ha^−1^ m^−1^, and landward marine mangrove soils (cf. UM1) 480–503 Mg ha^−1^ m^−1^. Fujimoto et al. (1999) found SOC stocks of 544–682 Mg ha^−1^ m^−1^ under a Micronesian marine mangrove forest, which is in accordance to our observations as well. Donato et al. (2012) reported SOC stocks under marine mangroves at the islands Yap and Palau of around 465 Mg ha^−1^ m^−1^ in both cases. Another study of the authors dealing with estuarine mangroves found SOC stocks ranging from 1000 to 1200 Mg ha^−1^ based on a sampling to 3 m depth, which equates to 330–400 Mg ha^−1^ m^−1^ for the first meter of soil (Donato et al. 2011). Murdiyarso et al. (2015) found generally high SOC stocks of 1083 Mg ha^−1^ based on 2 m soil depth in Indonesian mangroves. This equates 542 Mg ha^−1^ m^−1^, a magnitude we only observed for marine mangrove soils. In contrast to this, SOC stocks of the Sundarban mangroves (estuarine type) were estimated to be relatively low with 38–87 Mg ha^−1^ m^−1^ based on 30 cm sampling depth (Ray et al. 2011). Regarding the unique control factors of SOC stocks, it must be considered that estuarine and marine mangrove soils differ much in bulk density as well as in OC contents. Estuarine mangrove soils had bulk densities of up to four times higher than those of marine mangrove soils (0.33–0.80 g cm^−3^ and 0.18–0.27 g cm^−3^, respectively), while carbon concentrations in soil were up to 25 times higher in the soils of marine mangroves (ranging from 11 to 85 mg SOC g^−1^ and 170 to 260 mg SOC g^−1^, respectively). The fact that marine mangroves reveal higher SOC stocks despite their low bulk density of the soil makes the OC concentration the most prominent control factor. We can conclude that our data on mangrove SOC stocks are consistent with previously published data revealing a wide range with high SOC stocks for marine mangroves and lower SOC stocks for estuarine mangroves. Concerning their comparison, we suggest the conversion to uniform soil depths (e.g., 1 m) because the data are often referred to the total soil depth or the maximum sampling depth.

Considering degraded mangroves, it has been reported contradictory whether or not degradation has an impact on SOC or not. Sanders et al. (2014) found higher sedimentation of allochthonous nonmangrove organic matter in degraded mangroves and due to this suggest higher organic matter accumulation in degraded mangroves, although the role of a degraded hinterland yielding high erosion, thus sedimentation rates, was not discussed. A survey of Caribbean mangroves showed no differences in sedimentation rates but higher SOC contents in undegraded mangroves. (Granek and Ruttenberg 2008). In accordance with the latter, we observed the smallest SOC stocks of all sampled plots under degraded mangroves, concluding that degradation has a decreasing impact on mangrove SOC stocks.

Concerning the different SOC stocks between marine and estuarine mangroves, it might seem likely that the marine soils accreted over a longer time, whereas the estuarine soils are being dispersed and eroded. However, the available data of the radiocarbon age suggest that the undegraded marine soils are younger than the undegraded estuarine soil. Besides the pronounced differences in SOC stocks between the different mangrove types, the land‐to‐sea gradient in SOC stocks observed for each undegraded mangrove type (marine and estuarine mangroves) suggests additional controlling factors of SOC stocks (Figs. 3, 4).

In order to understand the biogeochemical triggers on the formation of the SOC pool, addressing the interplay between nutrient limitation gradients along the systems and decomposition of organic matter is crucial. A salinity gradient could be excluded to control the SOC stocks in our case, as salinity formed two independent clusters with no further correlations to SOC. Different tidal exposure and drainage might also exert an influence on root growth and thus OM formation. However, as the tidal range at UM with the largest OC stocks is intermediate and comparable to tidal ranges in the mangrove areas with the highest and the lowest SOC stock (UM and DE), this variable cannot explain different OC stocks.

As mangroves are known to be especially effective in the resorption of nutrients from leaves prior to litter fall (Rao et al. 1994; Hörtensteiner and Feller 2002), high C/N ratios of litter input to soil occur. We found C/N ratios of fresh leaves of up to 81 and of fresh roots of up to 143 which supports this finding (Table 3). Thus, comparably high C/N ratios in mangrove soils are the result. Indeed, it was shown in a litterbag experiment with 1 mm^2^ mesh size by Bosire et al. (2005) that coarse mangrove litter is decomposed by the mesofauna already within the first few weeks, resulting in stable C/N ratios of around 40, which still indicates a hampered microbial decomposition. This is similar to the C/N ratios observed for the topmost meter of marine mangrove soils in this study, but much higher than in their estuarine counterparts (Fig. 5A). Therefore, it can be concluded that the litter quality is one control factor of the SOC stocks. The low decomposability of litter imposes additional N limitation for marine mangroves, which might be overcome via symbiont‐mediated N_2_ fixation, an energy‐intensive method to adapt to specific N limitations (Sengupta and Chaudhuri 1991; Holguin et al. 2001; Bashan and Holguin 2002; Reef et al. 2010). Our dataset suggests that this additional pathway of N input is especially used by marine mangroves and only to a lower extent by estuarine mangroves. Firstly, an increase in the C/N ratio with soil depth in case of marine mangroves (UM1, Fig. 5A) could be a possible indication for an additional N source at the soil surface beside the organic matter itself. Secondly, *δ* ^15^N values of marine mangrove soils (UM 1–4, Fig. 5C) are close to 0‰, thus very close to the *δ* ^15^N ratio of air, which is a strong hint for dominating N_2_ fixation (Fogel et al. 2008). In contrast to this, estuarine mangrove soils exhibited clearly positive *δ* ^15^N values, indicating that pathways of N acquisition dominate which underlie stronger isotope fractionation than N_2_ fixation (DE 1–4, UE 1–4, Fig. 5C). Therefore, more intensive N recycling or additional N sources like nonmangrove plant litter with smaller C/N ratios or estuarine N transport from the hinterland has to be taken into account to explain higher decomposability of OC in estuarine mangrove soils. Indeed, we observed the lowest plant C/N ratio of around 19 (*Derris trifoliata* and *Acanthus ilicifolius*, Table 3) at the plot with the smallest SOC stock among all studied sites, where a pure pioneer plant community replaced the climax mangrove vegetation (DE1, Figs. 3, 5A). This, in the context of generally smaller SOC stocks in the whole degraded estuary (DE), suggests that mangrove degradation causes SOC stock depletion by accelerating SOC turnover rates by providing alternative organic matter sources with smaller C/N ratios via a community shift in vegetation.

Nevertheless, these differences in community composition are not the only reason for smaller SOC stocks in estuarine mangroves, as also the undegraded estuarine mangroves (UE 2–4) showed generally small SOC stocks that are decreasing along the land‐to‐sea gradient (Fig. 3). This might be attributable to the influence of organic matter sources from the hinterland to estuarine mangrove soils, as indicated by the *δ* ^13^C values of the respective plots. While marine mangrove soils (UM 1–4) show relatively uniform *δ* ^13^C values of around −28‰, which is similar to the fresh organic material of the mangroves growing there and additionally indicates a lower turnover of organic matter (Table 3), the *δ* ^13^C values of estuarine mangrove soils spread over a wider range and differ to the local input sources (Fig. 5B, Table 3).

**Figure 6:**
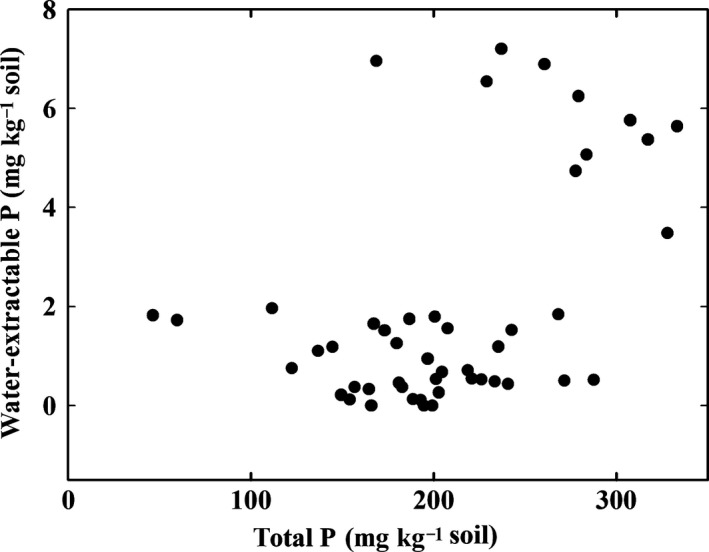
Water‐extractable P versus total P of mangrove soils from all stations. A poor correlation of both parameters suggests a P source other than the soil itself.

Another important plant nutrient next to N is P, which behaves contrarily to N in our study. Compared to the estuarine mangrove soils, marine soils exhibit far higher concentrations of water‐extractable, thus, plant‐available P. Organic matter itself is unlikely as the source of water‐extractable P, as there was no correlation between the total P and the water‐extractable P of the soil observed (Fig. 6). This suggests seawater as the primary source for the water‐extractable P in case of the marine mangroves. Available data for the Molucca Sea report PO_4_ concentrations of about 3.4 mg m^−3^ (Reid and Mantyla 1994). We assume this low concentration to be enough, because the mangroves are constantly supplied with fresh seawater by the diurnal tides. It is known as well that the water column of shallow coastal embayments holds up to the twentieth fraction of the P stock of the standing biomass of adjacent mangrove forests (Eyre and McKee 2002), which is potentially entering the marine mangrove soils via the diurnal input of fresh seawater by the tides.

In case of estuarine mangroves, where the P supply from the sea is decreasing as indicated by smaller water‐extractable P (Fig. 5D), the hinterland can be ruled out as a possible P source as also the water‐extractable P concentrations of the river sediments were much lower than those of the corresponding mangrove soils (Fig. 5D). This conclusion is additionally consistent with the general idea of P‐limited terrestrial tropical ecosystems (Vitousek 1984). The higher P concentrations of seaward compared to landward oriented mangroves as observed for the undegraded mangrove ecosystems in our study were found as well in a study on root biomass of mangroves along a land‐to‐sea gradient (Adame et al. 2014). Mangroves prone to the sea had larger root biomass due to higher contents of plant‐available P as compared to the corresponding inland mangroves. Castañeda‐Moya et al. (2013) found the contrary effect concerning root biomass, although the increasing P gradient from land to sea was likewise observed. We conclude that marine mangroves are well supplied with P from the ocean, whereas estuarine mangroves suffer landwards from P limitation due to diluted ocean water. Therefore, the mangroves at rather P‐limited sites depend on the higher SOC turnover, which is facilitated by the lower C/N ratios of the respective sites, to cover their P demand.

Due to the regional approach of our study, we could identify two scales of N and P colimitation leading to land‐to‐sea gradients in SOC stocks. The first scale is the different functional traits of the mangrove ecosystems in order to cope with this colimitation:

1. In marine mangroves, the relatively high concentrations of freely available P make N the limiting nutrient. This leads to higher resorption of N from mangrove leaves, as shedding of leaves with a low C/N ratio would impose an unnecessary waste of N by the plant. A waste of N has to be furthermore avoided, as this N has to be additionally acquired at high energy costs for the plant by symbiotic N_2_ fixation from air. The result is an accumulation of organic matter over time, as decomposition of organic material is neither promoted by available N nor needed for P supply of the mangrove, which results in larger SOC stocks in inland direction, as the mangroves propagate toward the sea.
2. Undegraded estuarine mangroves are constrained due to their P limitation. Hence, their P demand is likely covered via an increased decomposition of organic matter, which is further facilitated by the low C/N ratios in addition to the N sources from the hinterland.
3. These effects are furthermore strengthened if estuarine mangroves are degraded, because invading pioneer plants deliver litter with low C/N ratios.

**Figure 7:**
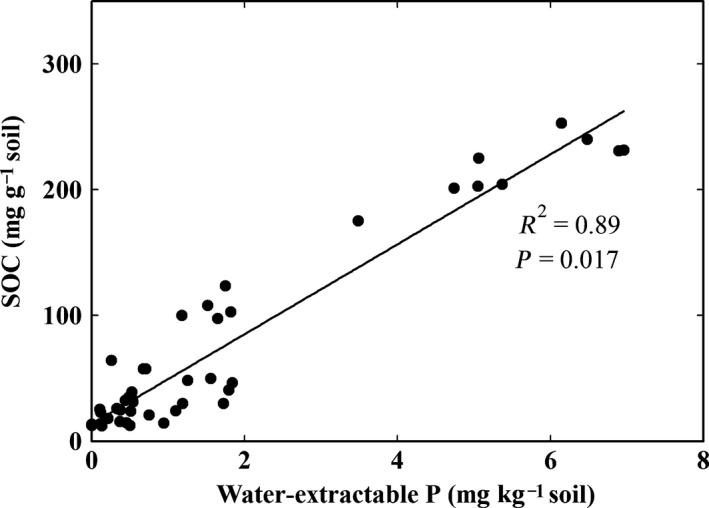
Soil organic carbon (SOC) concentration versus water‐extractable P of mangrove spoils from all stations. The good correlation suggests P to be a main control factor of SOC stocks.

The second scale is that, despite the differences in functioning at the ecosystem level, the concentration of water‐extractable P alone can explain SOC stocks over the whole region (*R* ^2^ = 0.89, *P* = 0.017, Fig. 7). Therefore, the availability of freely available P seems to be the most important driver of the SOC stocks in all mangrove soils, regardless of their marine or estuarine nature.

As the outcome of this study, we could not reject our initial hypothesis, but specified it functionally by likewise broadening its applicability in the way that P limitation governs biogeochemical fluxes in mangrove ecosystem across all scales and functional traits. Our findings may provide viable and easy‐to‐use proxies to estimate carbon stocks for PES and REDD schemes: the relative distance to the sea, the knowledge of the marine or estuarine nature of the mangrove ecosystem, and, if available, the water‐extractable P concentration of the soil already allow for sufficiently accurate estimations of SOC stocks in Indonesia and likely the whole Indo‐pacific region.

## Conflict of Interest

None declared.
